# The Effects of a Cultivar and Production System on the Qualitative and Quantitative Composition of Bioactive Compounds in Spring Wheat (*Triticum* sp.)

**DOI:** 10.3390/molecules29174106

**Published:** 2024-08-29

**Authors:** Iwona Kowalska, Sylwia Pawelec, Łukasz Pecio, Beata Feledyn-Szewczyk

**Affiliations:** 1 Department of Biochemistry and Crop Quality, Institute of Soil Science and Plant Cultivation-State Research Institute, Czartoryskich Str. 8, 24-100 Pulawy, Poland; spawelec@iung.pulawy.pl (S.P.); lpecio@iung.pulawy.pl (Ł.P.); 2 Department of Systems and Economics of Crop Production, Institute of Soil Science and Plant Cultivation-State Research Institute, Czartoryskich Str. 8, 24-100 Pulawy, Poland; bszewczyk@iung.pulawy.pl

**Keywords:** UPLC-DAD-MS, phenolic acids, alkylresorcinols, TLC-DPPH^•^, natural products, hulled wheat species, hull-less wheat species

## Abstract

Spelt *Triticum aestivum* L. subsp. *spelta* (cv. Wirtas), einkorn *Triticum monococcum* L. (cv. Samopsza) and emmer *Triticum dicoccum* Schrank (Schuebl) (cv. Płaskurka biała and Płaskurka ciemna) spring wheat cultivars were analyzed and compared to common wheat *Triticum aestivum* L. subsp. *aestivum* (cv. Harenda, Kandela, Mandaryna, Serenada, Goplana, Kamelia, Nimfa, Rusałka, Struna, Zadra) cultivated in an organic production system. Moreover, the performance of four common wheat cultivars (cv. Harenda, Kandela, Mandaryna, Serenada) grown in organic, conventional and integrated production systems were compared. The UHPLC-DAD-MS and TLC-DPPH^•^ analyses of specific substances (phenolic acids and alkylresorcinols) were evaluated to ascertain the potential of spring wheat cultivars for promoting human health and suitability for cultivation in an organic production system. The highest yield was observed for the *T. aestivum* L. subsp. *aestivum* (modern hull-less) cv. Nimfa (4.45 t/ha), which also demonstrated the lowest resistance to *Fusarium* spp. infection. Among the contemporary hull-less cultivars, cv. Mandaryna and cv. Harenda exhibited the highest resistance to this pathogen (2.4% and 3.7% of grains infected by *Fusarium*, respectively), while simultaneously displaying the highest organic phenolic acid content (900.92 and 984.55 µg/g of the grain) and the highest antioxidant potential. It is noteworthy that the cereal hulls of *T. monococcum* L. (old hulled) (cv. Samopsza) exhibited a markedly elevated content of phenolic acids (approximately 4000 µg/g of the grain). This may have contributed to the reduced incidence of *Fusarium* infection (9.3% of grains infected) observed in the grains of this cultivar. Furthermore, the hulls proved to be a rich source of phenolics with high antioxidant activity, which is beneficial for human and animal health.

## 1. Introduction

The dynamically growing interest of consumers, farmers and food producers in hulled cereal crops is due to their healing potential and greater resistance to abiotic stress, including increasingly common drought conditions. The cultivation of these species presents an opportunity for preserving agricultural biodiversity and valuable genetic material adapted to the local soil and climate. Currently, hulled cereals, including spelt, emmer and einkorn wheat, are the most commonly grown of the ancient wheat cultivars in Poland. 

The cultivars of hulled wheat are recognized for their valuable grain, biochemical composition and potential health benefits. Hulled wheat species are a promising alternative source of high-quality materials for the agri-food industry, especially in the context of modern agricultural production [1]. This species has more protein, minerals, fiber, lipids and vitamins than common wheat, and its amino acid composition is more appropriate [2]. Spelt wheat (*Triticum aestivum* L. ssp. *spelta*) is an old European wheat species that has gained attention for its nutritional benefits [3]. For people with diabetes [4] and people who are overweight or obese [5], spelt wheat is a better alternative to common wheat. Compared to common wheat grain, spelt grain is richer in minerals and protein and is characterized by higher digestibility, as well as higher biological and nutritional value [6]. The high content of bioactive substances means that products made from einkorn wheat (*T. monococcum* L.) can be called functional and even health-promoting food. They are rich in protein, up to 50% more than modern (hull-less) wheat, which is a very beneficial composition in terms of health [7]. Hulled wheats have a beneficial effect on the nervous system. This is because they contain amino acids such as phenylalanine, tyrosine, methionine or isoleucine, which are not found in common wheat. Spelt is an excellent source of fiber, iron, zinc, phosphorus, B vitamins and lutein, a powerful antioxidant that protects our eyesight from diseases. Emmer wheat (*T. dicoccum* Schrank (Schuebl)), a traditional part of human diets, boasts disease resistance and good grain quality, with high protein content ranging from 17.0 to 22.5%, along with high carotenoid levels. Additionally, it has excellent properties for producing biscuits and yeast-free bakery items [8]. Whole wheat flour is a nutritious food source due to its high crude protein content compared to modern cultivars. Additionally, it is a vital source of dietary fiber and is abundant in essential minerals, such as copper, magnesium, phosphorous, zinc, potassium and manganese [9]. A comparative analysis of bioactive components (i.e., dietary fiber, phenolic acids, folates) in ancient and modern wheat varieties revealed differences between the two groups. It was shown that emmer and einkorn had two to three times more carotenoid lutein than common wheat, but durum wheat showed comparable lutein content [10]. With genetic advancements, modern cultivars have lost natural adaptation characteristics in favor of improved quantitative and qualitative traits. The most desirable cultivars are those that have high levels of bioactive compounds, a high yield potential and are resilient to fungal diseases and infestation. 

Very important components of wheat grains are both alkylresorcinols (ARs) and phenolic acids (PAs), which are secondary metabolites having antioxidant properties. Including biologically active compounds in one’s daily diet may potentially increase resistance to heart disease, cancer and atherosclerosis [11]. This is especially relevant during times of growing nutritional awareness. Consumers seek and desire food products containing these compounds. ARs are viewed as prospective bioregulators of immunological and metabolic processes [12], in addition to serving as therapeutic adjuvants for anticancer and antimicrobial treatments [13]. The characteristics of ARs align with public health advice advocating for whole-grain consumption to prevent chronic diseases. An extensive comprehension of the chemical structure, physical and chemical properties and biological functionalities of ARs, may lead to their use as medicinal, health-enhancing agents or within the cosmetic industry [13,14].

The objective and novelty of this study were the comparison of spelt, einkorn, emmer and common spring wheat performance using multi-criteria analysis. This analysis included biometric data, grain yield, plant infestation by pathogens and the occurrence of *Fusarium* spp.; natural product composition in spelt, einkorn and emmer spring wheat were analyzed and compared to common wheat. Chemical analysis was performed using a chromatographic analysis of phenolic acids, ARs and thin-layer chromatography in combination with biodetection (TLC-DPPH^•^). Furthermore, the research aimed to compare the grain quality and yield of four modern wheat cultivars cultivated across various crop production systems (integrated, conventional and organic). The results could aid in directing the breeding or engineering of plants with improved concentrations of these compounds, while maintaining higher yield cultivars. 

## 2. Results and Discussion

### 2.1. The Results of the Organic Production System

#### 2.1.1. Biometric Analyses and Grain Yield, Evaluation of Plant Infestation by Pathogens and *Fusarium* spp. Occurrence

Among common wheat cultivars, Nimfa and Goplana were the best yielded cultivars, which is related to the biggest density of ears in the canopy (Table S1). In addition, cv. Goplana and cv. Serenada were characterized by a high thousand kernel weight (Table S1), which was connected to the lowest level of fungal pathogens infestation/the best health status (Table S2). The cause for low yields of cv. Kamelia and cv. Zadra was their high infestation by fungal pathogens and the small density of ears in the canopy (Tables S1 and S2). The dominant pathogens infecting wheat leaves in 2017 were *Drechslera tritici-repentis* (Died.) Shoem. and *Puccinia recondita* Dietel & Holw in 2018. Despite the very good health status of cv. Kandela (the smallest fungi infestation—8.61%), it was characterized by a small number of ears in the canopy and a small thousand kernel weight, which resulted in a low yield of this cultivar.

The old wheat cultivars have a lower yield potential than the common wheat cultivars (Table S1), although they have valuable biochemical composition and, as a result, health potential, as described in the following subsections. Hulled wheat gave 30–56% lower yields compared to common and durum wheat, even when cultivated with advanced technology [1]. Emmer and spelt maintained appropriate technological parameters even despite lower yields. It is only the high-gluten emmer cultivar which may present a limitation in its use as a raw material in some food production processes.

**Figure 1:**
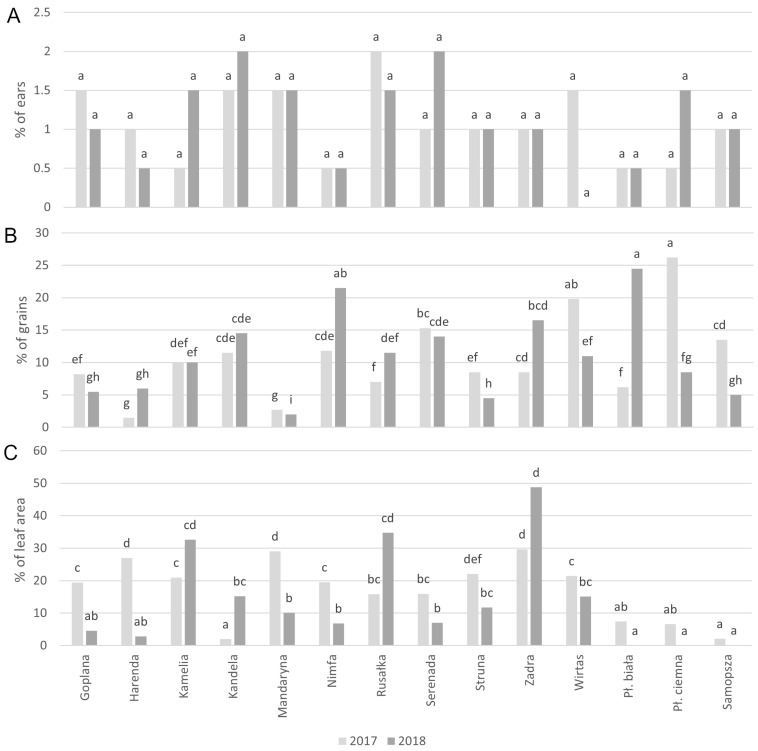
Infestation of ears (**A**) and grains (**B)** by *Fusarium* (different letters indicate significant differences (*n* = 12) according to χ^2^ test at *p* ≤ 0.05) and leaves (**C**) by total fungal pathogens of spring wheat cultivars in organic production system (different letters show significant differences (*p* ≤ 0.05) after a post hoc Tukey’s honest significance difference (HSD) test).

The occurrence of *Fusarium* spp. on the ears (Figure 1A) and the infestation of the grains (Figure 1B) of spring wheat cultivars were assessed, too. In 2017–2018, ear fusariosis occurred sporadically (Figure 1A). When analyzing the suitability of the examined spring wheat cultivars for the organic production system, a slight variation was observed (the percentage of infected ears of individual cultivars ranged from 0 to 2.0% and these differences were not statistically significant). Grain colonization by fungi of the genus *Fusarium* did not reflect the severity of ear fusariosis (Figure 1B). In both years of the study, the average infestation by *Fusarium* spp. was at a similar level (10–11% of infested grains). Mycological analysis also showed a large variation in grain infestation by *Fusarium* spp. of the tested cultivars. On average from 2017 to 2018, the least infested kernels were cv. Mandaryna and cv. Harenda (2.4–3.7%) and the most infested were cv. Nimfa and cv. Serenada among the common wheat cultivars (14.7–16.6%), with cv. Wirtas, cv. Płaskurka biała and cv. Płaskurka ciemna among the hulled wheat cultivars (15.3–17.3%). The hulled cultivars emmer and einkorn were characterized by about six times lower infection by fungal pathogens in comparison to the modern cultivars of common wheat (Table S2, Figure 1C). 

#### 2.1.2. Quantitative and Qualitative Analysis of PAs in Grain and Husk

**Figure 2:**
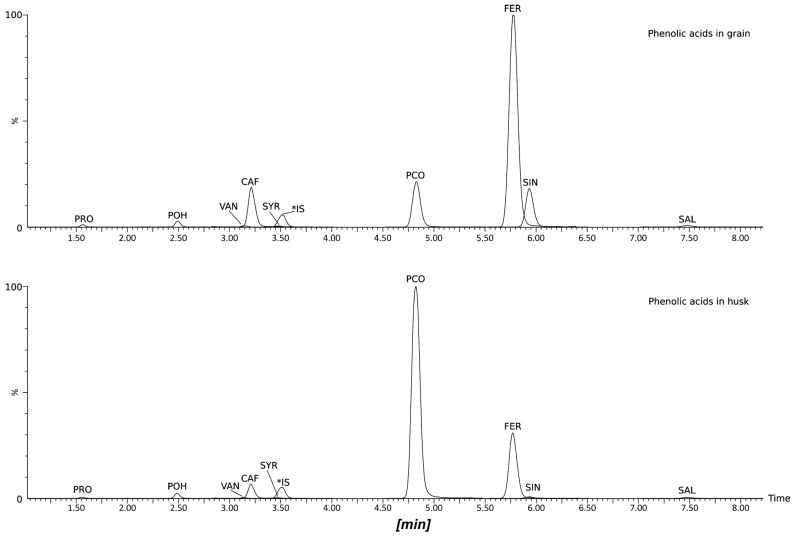
UPLC-MS/MS chromatograms in MRM mode of cv. Wirtas (2018) sample. Peak annotations: PRO = protocatechuic acid, POH = *p*-OH-benzoic acid, VAN = vanillic acid, CAF = caffeic acid, SYR = syringic acid, *IS = Internal Standard (*m*-OH-benzoic acid), PCO = *p*-coumaric acid, FER = ferulic acid, SIN = sinapic acid, SAL = salicylic acid.

**Figure 3:**
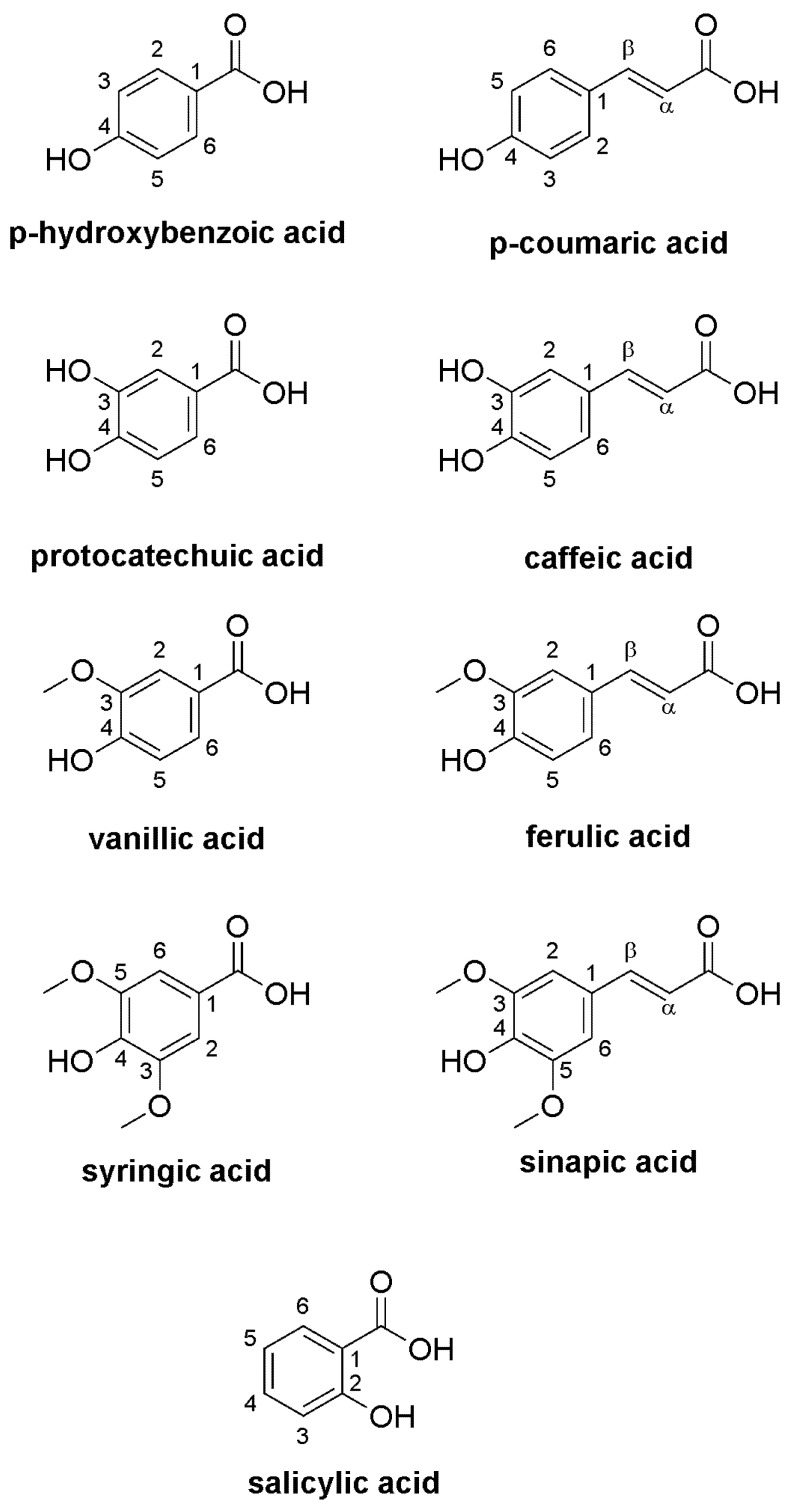
Chemical structures of spring wheat phenolic acids.

The UPLC-DAD-MS method was employed to analyze the PA content of wheat extracts (Figure 2). The following compounds were identified and quantified in the grain of hull-less spring wheat species (*T. aestivum* L. subsp. *aestivum*) as well as the grain and husk of hulled spring wheat species (*T. aestivum* L. ssp. *spelta*, *T. dicoccum* Schrank (Schuebl) and *T. monococcum* L.): protocatechuic, *p*-OH-benzoic, vanillic, caffeic, syringic, *p*-coumaric, ferulic, sinapic and salicylic acids (Figure 3). 

**Figure 4:**
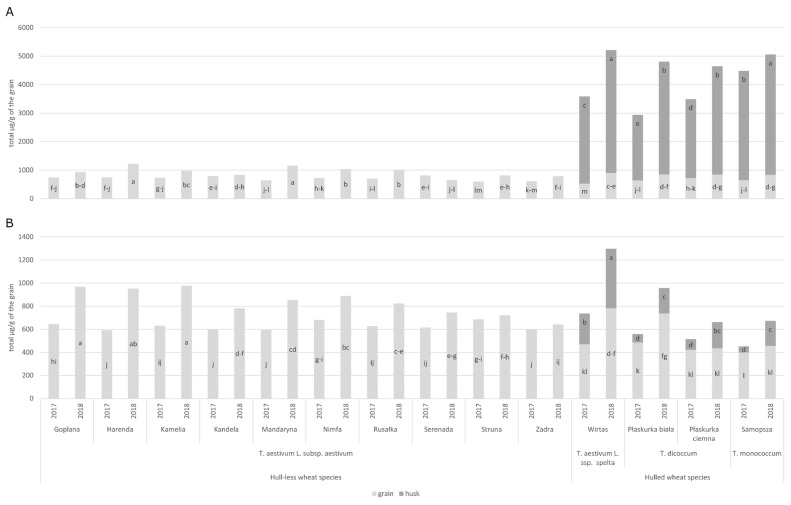
An average concentration of total phenolic acids (**A**) and alkylresorcinol derivatives (**B**) of hull-less and hulled wheat cultivars cultivated in organic production system. Different letter indicates significant differences (*p* < 0.05); statistical analysis was performed separately for grain and husk.

A comparison of *T. aestivum* L. subsp. *aestivum* cultivars indicates that PAs exhibited a high degree of variability in concentration (Table S3). Wheat cultivars grown in 2018 had a significantly higher PA content (mean 919.01 μg/g of the grain) compared to 2017 (mean 690.6 μg/g) (Figure 4A).

This may be attributed to meteorological conditions during the growing season. The average monthly temperature between April and August was higher in 2018 than in these months in 2017. Furthermore, in June and July 2018, the total precipitation was higher compared to 2017 (Table S4). Pu et al. [15] demonstrated that high air temperature and radiation intensity are associated with high bioactive content and the antiradical activity of grains. Research by Fernandez-Orozco et al. [16] indicated that a range of environmental stresses, including high temperatures during grain filling and excess water, can induce an increase in phenolic content. Similarly, Shamloo et al. [17] observed an increase in free PA content in wheat grain with increasing temperature during plant growth. The content of PAs per gram of grain, in 2017 and 2018, ranged from 524.5 to 815.2 µg/g and from 660.1 to 1223.3 µg/g, respectively (Table S3).

A quantitative and qualitative analysis of PAs in the grain and husk of hulled wheat cultivars revealed notable differences. PAs content was significantly affected by wheat species and crop year, similar to the study by Zrckova et al. [18]. The total PAs content was significantly higher in 2018, compared to 2017 (Figure 4A). The average PA content of the husk was more than five times higher than on the grain. Both in the grain and in the husk, the highest content of the tested compounds was observed in cv. Wirtas (*T. aestivum* L. ssp. *spelta* species) and cv. Samopsza (*T. monococcum* species). Ferulic acid was the predominant PA in the grain, while *p*-coumaric acid predominated in the husk, in all the spring-hulled wheat cultivars (Table 1 and Table S4). Barański et al. [7] observed that the total free PA content was higher in spelt (*T. aestivum* L. ssp. *spelta*) at a value of 599.8 µg/g DM, while in emmer wheat (*T. dicoccum* Schrank), it was 590 µg/g DM. Similarly, the bound PA content in spelt was 564.6 µg/g DM, while in emmer it was 555.4 µg/g DM. Spelt wheat exhibited the highest free *p*-hydroxybenzoic acid content (1.96 µg/g DM) and the highest syringic acid content (3.25 µg/g DM). The lowest content was observed for caffeic acid (0.58 µg/g DM), *p*-coumaric acid (1.10 µg/g DM) and sinapic acid (1.52 µg/g DM). The free salicylic acid content of emmer wheat was found to be lower, with a value of 0.99 µg/g DM, while the syringic acid content was 2.23 µg/g DM. Similar results were observed in other research, in which free phenolic content ranged from 164.25 to 271.05 mg GAE/100 g DW and the bound phenolic content was between 182.89 and 565.62 mg GAE/100 g in spring breeding lines of purple wheat. The total phenolic value ranged from 352.65 to 771.83 mg GAE/100 g [19]. In the research of Li et al. [20], the average total PAs content of spring wheat was 612 μg/g DM. The average level of total PAs in einkorn cultivars was 615 μg/g DM, whereas that in the *T. monococcum* was slightly higher (779 μg/g DM). The lowest average level (579 μg/g DM) of the total PAs was observed in the spelt cultivars. In a report published by Skrajda-Brdak et al. [21], it was demonstrated that the total amount of PAs present in spring durum wheat ranged from 420 mg/kg DM to 838 and 1060 mg/kg DM. Of all the phytochemicals present, the major phytochemicals were PAs, which accounted for 43% of the total. The most abundant phytochemical was ferulic acid, with a range of 367.4 to 1007.6 mg/kg DM. The lowest amount was observed for syringic acid, with a range of 1.9 to 12.3 mg/kg DM. 

Ferulic acid is one of the most abundant PAs in wheat grain [22]. In our research, it was found to be the main constituent in all cultivars, regardless of the year and crop production system. The mean content of this compound in individual cultivars ranged from 70.44% (cv. Płaskurka ciemna, in 2017) to 88.36% (cv. Kandela, in 2017) of the total PA content in grain. The highest content of this compound was observed in cv. Harenda, grown in 2018, with a value of 1062.98 µg/g of the grain (Table S3). Our results were consistent with the study by Mpofu et al. [23], who showed a high genetic variability (cultivar-dependent) in ferulic acid content. However, its content reported in the present study was found to be higher than that previously published by Mpofu et al. [23], who indicated that the content in different varieties ranged from 371 to 441 µg/g. The results of the study were compared with those of an earlier study by Żuchowski et al. [24]. It has been demonstrated that the most prevalent PA in wheat grain was ferulic acid, accounting for 85.3–89.3% of the total phenolic acids. Additionally, lower amounts of other acids, including *p*-coumaric, *p*-OH-benzoic, salicylic and syringic acids, were also present in Polish wheat grain. The predominant PAs detected by Li et al. [20] in the spring wheats were ferulic, syringic, vanillic and sinapic acids. The average ferulic acid content was 84% in the spring wheat cultivars. As shown in the study by Abdel-Aal et al. [25], the concentration of ferulic acid was found to differ significantly in the mature grain of spring wheat cultivars known to have a range of tolerance to the *Sitodiplosis mosellana*. Differences in the content of this acid were correlated with the level of infestation of the cultivars.

Our research has shown that in the husk, ferulic acid was not the most prevalent PA, with concentrations ranging from 36.71% (cv. Samopsza, in 2017) to 51.09% (cv. Wirtas, in 2018) of the total PA content. Instead, *p*-coumaric acid was the dominant PA. This compound exhibited a range of concentrations, from 45.56% (cv. Wirtas, in 2017) to 57.02% (cv. Samopsza, in 2017). The husk of cv. Wirtas, grown in 2018, exhibited the highest content (4318.53 µg/g of the grain) of this compound. 

#### 2.1.3. Antiradical Activity of PAs Fraction

It is believed that phenolic compounds, especially phenolic acids (PAs), are one of the main factors influencing the antioxidant activity of cereal grains [26]. Chen et al. [27] considered phenolic acids to be natural antioxidants with potential health advantages. The results of this research demonstrated that a lot of compounds with antiradical activity are present in the husks of hulled wheat species—*T. aestivum* L. ssp. *spelta*, *T. dicoccum* and *T. monococcum* (Table S3)—which may be of great importance to the baking industry. The husk of the cv. Wirtas, grown in 2018, had the highest antiradical activity (average 0.892 in relation to caffeic acid standard), as well as cv. Samopsza (mean 0.885) and cv. Płaskurka ciemna (mean 0.878). The high antiradical activity of the husk is due to the attendance of *p*-coumaric, vanillic and ferulic acids content (Table S3). Wheat bran is a good source of PAs, which importantly contributes to the whole antioxidant activity of wheat. Moreover, they can inhibit lipid oxidation catalyzed by iron or peroxyl radicals [28]. Ferulic acid is believed to be a main factor influencing the total antioxidant activity of wheat. Following the DPPH scavenging process, several semiquinones derived from ferulic acid may undergo dimerisation, resulting in the enhancement of their antiradical activity. The grain of all the tested cultivars showed significantly less activity than the husk. Statistically significant differences (*p* < 0.05) in the antiradical activity of the grain of individual cultivars were found (Table S3). The antiradical activity of wheat grain is a result of the presence of *p*-coumaric, ferulic and caffeic acids content (Table S3). Among the grains of all hull-less wheat cultivars, cv. Harenda and cv. Mandaryna showed the highest mean antiradical activity in 2018 (0.296 and 0.262 with reference to standard, respectively). Caffeic acid exhibits greater antiradical activity due to the presence of additional conjugation in the propene side chain, which may facilitate electron delocalization through resonance between the propene group and the aromatic ring. Furthermore, ferulic acid with one methoxy group is more active than *p*-coumaric acid (containing one hydroxyl group) [27]. Our averaged phenolic acid activity results were higher than in previously published data. In the study of Kowalska et al. [29], the mean antiradical activity of winter wheat cultivars, grown in Poland, was 0.208.

#### 2.1.4. Identification and Quantification of Alkylresorcinols (ARs, Resorcinolic Lipids)

**Figure 5:**
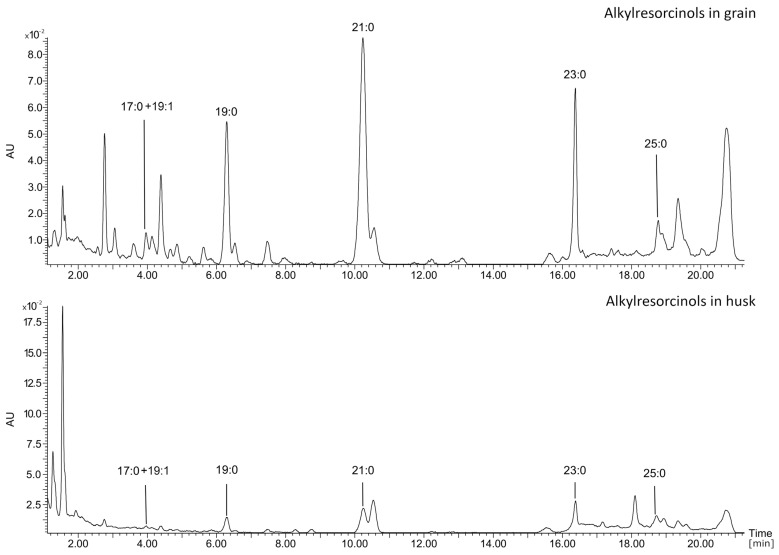
UPLC-PDA (λ = 275 nm) chromatograms of spectrum for all alkylresorcinols derivatives determined in the grain and husk of spring wheat cultivars; 5-*n*-heptadecylresorcinol (C17:0), 5-*n*-nonadecylresorcinol (C19:0), 5-*n*-nonadecenylresorcinol (C19:1), 5-*n*-heneicosylresorcinol (C21:0), 5-*n*-tricosylresorcinol (C23:0) and 5-*n*-pentacosylresorcinol (C25:0).

**Figure 6:**
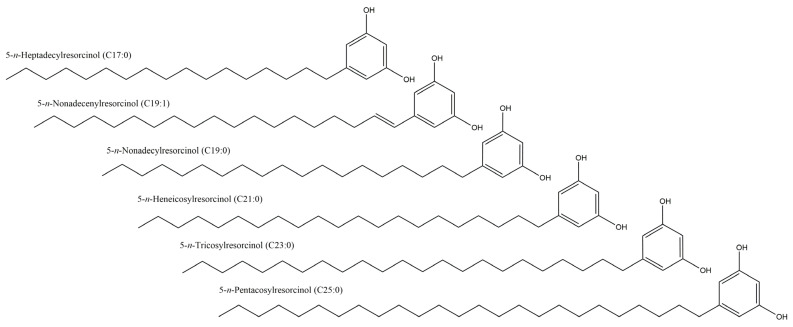
Chemical structures of 5-*n*-heptadecylresorcinol (C17:0), 5-*n*-nonadecylresorcinol (C19:0), 5-*n*-nonadecenylresorcinol (C19:1), 5-*n*-heneicosylresorcinol (C21:0), 5-*n*-tricosylresorcinol (C23:0) and 5-*n*-pentacosylresorcinol (C25:0).

The composition of ARs in spring wheat cultivars was analyzed using the UPLC-PDA-MS/MS technique **(**Figure 5), which enabled the identification of six AR derivatives: 5-*n*-heptadecylresorcinol (C17:0), 5-*n*-nonadecylresorcinol (C19:0), 5-*n*-nonadecenylresorcinol (C19:1), 5-*n*-heneicosylresorcinol (C21:0), 5-*n*-tricosylresorcinol (C23:0) and 5-*n*-pentacosylresorcinol (C25:0) (Figure 6). As illustrated in Figure 5 and Table S5, the homologues C21:0 and C19:0 were the most prevalent in wheat grain, while in the husk, C25:0 and C21:0 were the most prevalent. The concentration of alkylresorcinols in *T. aestivum* L. subsp. *aestivum* exhibited notable variation (at the level of *p* < 0.05) between both the different cultivars and across different years of growth ranging from 590.96 μg/g (cv. Harenda in 2017) to 977.43 μg/g (cv. Kamelia in 2018) (Figure 4B, Table S5). In previously published studies by Skrajda-Brdak et al. [30] in Polish wheat grains, the average AR content was about 723 μg/g, as well as 800 μg/g in the study by Kulawinek et al. [31]. Other studies on Polish spring wheat cultivars showed alkylresorcinol contents ranging from 471 to 995 µg/g (average 680 µg/g) [32]. These differences may be due to variability in environmental, cultivar and analytical conditions [33].

The ARs abundance of the grain and husk of hulled wheat cultivars was found to be significantly higher in 2018 compared to 2017 (Table S5). The cv. Wirtas (*T. aestivum* L. ssp. *spelta* species) had the highest ARs total content in both the grain and husk in 2018 (782.1 and 515.3 μg/g grain, respectively) (Figure 4B). This content was more than twice as high as in the husks of the other hulled wheat cultivars analyzed (with the only exception of the ARs content in the grain of the cv. Płaskurka biała). This value is also significantly higher than that reported by Andersson et al. [34], who determined the AR value for five spelt cultivars (mean 605 µg/g DM) and ten durum wheat cultivars (mean 399 µg/g DM).

Another study on old cultivars of *T. monococcum* and *T. dicoccum* demonstrated elevated levels of ARs, with concentrations exceeding 450 mg/kg in wholemeal flour and bread. GC-MS analysis revealed that the predominant components were a mixture of 5-*n*-alkylresorcinols with a side chain between C15 and C25, predominantly C19 and C21 [14]. The grain from hull-less wheat species exhibited a higher average content of ARs than the grain from hulled wheat species (Figure 4B). Additionally, the husk content of the C17:0 as well as the C19:1 derivatives was present at the lowest levels or below the limit of quantification (Table S5). The discrepancies in alkylresorcinol composition between cultivars grown in disparate years may be attributed to the influence of environmental factors, such as weather patterns and the varying responses of the cultivars to the environmental stressors that occurred [33].

#### 2.1.5. Antiradical Activity of ARs

The TLC-DPPH^•^ assay in combination with ImageJ software was used to assess the antiradical activity of resorcinolic lipids. As a standard *α*-tocopherol was used. The results of the antiradical activity of the ARs are shown in Table S5. The grain of *T. aestivum* cultivars (hull-less wheat species) grown in 2018 had a significantly higher antioxidant activity (average 0.291 in relation to *α*-tocopherol’s activity) compared to 2017 (mean 0.219). Among the modern wheat cultivars, cv. Kamelia had the highest antiradical activity (mean 0.271 in relation to *α*-tocopherol’s activity), then cv. Goplana (0.269) and cv. Nimfa (0.268) (Table S5). This may have been influenced by these cultivars’ high total alkylresorcinol content, and mainly by the high C19:0 and C21:0 derivatives content. Analyses of the hulled wheat species showed that the grain had a significantly higher antioxidant activity compared to the husk. This was influenced by the significantly lower total ARs content in the husk. Of the tested cultivars, the highest activity was shown by the grain of the cv. Płaskurka biała (0.286) and cv. Wirtas (0.267) in the second year of the experiment.

The aliphatic side chain length and phenolic ring of an ARs molecule significantly impacted its antioxidant activity. It can be posited that the longer the alkyl chain, the stronger the antioxidant activity associated with the AR. The findings of this study are consistent with previous reports by Kowalska and Jędrejek [32] and Kowalska et al. [29], which indicate that ARs exhibit weak antioxidant activity against various tocopherols. Our results are also confirmed by studies by Kamal-Eldin et al. [35]. They showed that ARs are poor radical scavengers relative to different tocopherols based on the slow reaction rate and high molar ratio required to bleach the stable DPPH radical.

### 2.2. Comparison of Three Different Production Systems

#### 2.2.1. Quantitative, Qualitative Analysis and Antiradical Activity of PAs

**Figure 7:**
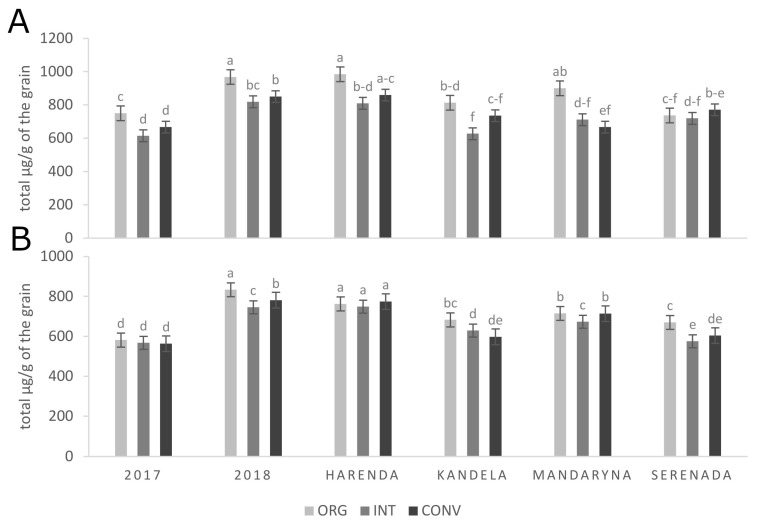
An average concentration of total phenolic acids (**A**) and alkylresorcinol derivatives (**B**) of four *T. aestivum* L. subsp. *aestivum* cultivars cultivated in different production systems (ORG—organic; INT—integrated and CONV—conventional). A different letter indicates significant differences (*p* < 0.05).

A two-year experiment comparing four modern wheat cultivars grown under three different crop production systems (organic, integrated and conventional) revealed significant differences in PAs content. This study demonstrated that the content of PAs is influenced by both the cultivar and years of experiment (Figure 7A). PAs concentrations were significantly different in organic, integrated and conventional wheat cultivars. Wheat cultivars grown under the organic production system in 2018 exhibited the highest total PA content, with the exception of cv. Serenada (Table 1). 

This may be due to the fact that increased PAs are an important defense factor for wheat plants growing under a wide range of stress conditions, including pathogen, insect and herbivore attacks, as well as the less selective herbicide treatments used in organic production systems [36]. Additionally, the application rate of fertilizer may have influenced the differences in the content of the tested compounds. Stumpf et al. [37] demonstrated in their study that non-fertilized wheat contained a greater concentration of soluble PAs in the grain compared to fertilized conditions. Pandino et al. [38] emphasize that the interaction between cropping systems and bioactive compound content varies by genotype. Previous research by Żuchowski et al. [24] demonstrated that phenolic acids concentrations were significantly different in organic and conventional wheat. Ferulic and *p*-coumaric acid levels, as well as total phenolic acid content, were higher in organic crops. In contrast, sinapic, *p*-hydroxybenzoic and vanillic acid concentrations in spring wheat were significantly higher in conventionally grown wheat.

Large differences were observed in the antioxidant activity of wheat grain between different production systems. Comparing the three production systems, samples in the organic system showed the highest antiradical activity, followed by conventional and integrated production systems. A significant positive correlation was observed between total phenolic acid content and total antiradical activity (Table S5).

#### 2.2.2. Identification, Quantification and Antiradical Activity of ARs

The production system, as well as the duration of the research, influenced the alkylresorcinols content in four modern spring wheat cultivars (Figure 7B). The highest ARs content was observed in 2018 for the organic production system (Table 2). The reduction in synthetic pesticides promotes the production of plant-specific substances that are a natural response to pathogen attacks due to the stimulating properties of environmental stress. As Andersson et al. [34] confirmed, organic cereal farming is associated with higher levels of plant metabolites, which is particularly interesting in the context of healthy foods. Takač et al. [39] examined both grain and bread from spring spelt wheat in various production systems (conventional and organic) and found that AN organic production system resulted in a significant increase in the ARs content (511.6 µg/g) compared to THE conventional production system (381.5 µg/g). For common wheat, this increase was relatively minor, from 439.9 to 454.9 µg/g.

Comparing the activity results for four modern cultivars grown in three farming production systems, it was shown that a comparative assessment of wheat cultivars grown in an organic system revealed that those cultivars exhibited the most notable activity (Table 2). The results of this study indicated that the antiradical activity of wheat extracts exhibited a positive correlation with the total amount of ARs. It correlates with our previously published data, which found a statistically significant effect of cropping systems on ARs antioxidant activity in both spring and winter wheat. The free radical scavenging activity of four spring wheat varieties and four winter wheat cultivars, which were grown under two different cropping systems, was contrasted [32].

## 3. Materials and Methods

### 3.1. Characteristics of Sites, Agronomic Practices, and Design of Experiment

The study with the cultivars of spring wheat was performed in the years 2017–2018 in the field experiment of the Institute of Soil Science and Plant Cultivation-State Research Institute (IUNG-PIB) in Osiny (Poland, Lublin voivodeship, N: 51°28′, E: 22°30′), on Haplic Luvisol soil with a texture of loamy sand. The soil exhibited a pH_KCl_ of 5.9 and a medium phosphorus (60.3 mg/kg) and potassium (104.0 mg/kg) content. The humus abundance was 1.6%. The spring wheat was grown in different crop production systems: organic (ORG), conventional (CONV) and integrated (INT) (Table S6). The compared systems differed in crop rotation, organic and mineral fertilization and crop protection against weeds, diseases and pests.

The experimental sites belong to a moderately continental climate zone. Meteorological conditions in the 2 years of the study are characterized in Table S4. In 2017, the temperature in the growing season was similar to the long-term average, whereas in 2018, the monthly temperatures were higher than the multiannual means. In both years of the study in June, the precipitation was about half as low as the long-term mean for this month, which could have influenced the wheat yield.

No synthetic fertilizers or pesticides were applied in the organic system. Only natural phosphorus (P) and potassium (K) fertilizers in the form of crude potassium salt or kainite, as well as compost, used one time in a crop rotation, under potato (30 t/ha), were applied. The conventional system included 3-field crop rotation: winter oilseed rape, winter wheat and spring wheat, where the crops were intensively cultivated, e.g., with the use of pesticides and synthetic mineral fertilizers (Table S7). The integrated system included a balanced mineral and organic fertilization, which was approximately 20–30% lower than in the case of the conventional system. The fertilization was applied according to the crop requirements and soil fertility. Nonchemical (mechanical) measures were used for crop protection, with only a small number of herbicides or other plant protection products applied with regard to harmfulness thresholds (Table S7). 

In each crop production system (organic, conventional and integrated), spring wheat was cultivated every year. Within each field of spring wheat, a split-plot experiment with cultivars was established in four blocks with the treatments randomized. In the organic system, 4 hulled and 10 hull-less cultivars of spring wheat were cultivated, whereas in the integrated and conventional systems, 4 hull-less cultivars were grown, the same as in organic ones, so we could compare them (Table S8). 

Soil tillage was carried out in the form of a traditional plow system in all crop production systems. Sowing treatments were conducted at the optimum time for the region, according to the rules of good agricultural practice. The area of each plot of replication measured 30 m^2^ for sowing and 25 m^2^ for harvest. Each cultivar was sown at the same sowing rate—450 grains/m^2^ in rows spaced 12 cm, with a depth of planting at 3.5 cm. Spelt wheat, emmer and einkorn were sown at 5 cm depth. Harvests were performed in the beginning of August in 2017 and 2018, at full ripeness stage (BBCH 89). 

### 3.2. Plant Material

The material for the study consisted of grain from 4 spring wheat species: common wheat *T. aestivum* L. subsp. *aestivum* (modern hull-less cv. Harenda, Kandela, Mandaryna, Serenada, Goplana, Kamelia, Nimfa, Rusałka, Struna, Zadra), spelt wheat *T. aestivum* L. subsp. *spelta* (L.) Thell. (modern hulled cv. Wirtas), two emmer cultivars *T. dicoccum* Schrank (Schuebl) (old hulled (cv. Płaskurka biała/white and Płaskurka ciemna/dark) and one cultivar of einkorn *T. monococcum* L. (old hulled cv. Samopsza). All of the cultivars were from an organic production system of two years’ experience; four of them (cv. Harenda, Kandela, Mandaryna, Serenada) were also from an integrated and conventional production system. The husks of spelt, emmer and einkorn cultivars were also analyzed like grains. Following the harvest, the grains were milled with an Ultra Centrifugal Mill ZM 200 (Retsch, Haan, Germany). Then, whole-grain flour with a middling granulation of 0.8 mm was obtained for use in chromatographic analysis. The obtained plant material was stored at the Department of Biochemistry and Crop Quality, Institute of Soil Science and Plant Cultivation-State Research Institute in Pulawy, Poland. 

### 3.3. Biometric Analyses and Grain Yield

The number of ears was determined from the area of 1 m^2^, in 4 repetitions, for each cultivar in the organic system. The harvest was performed with the use of a special plot harvester Wintersteiger for the whole plot area. Thousand kernel weight and grain yield were evaluated after harvest and calculated at a 15% moisture content. The grain from the harvester was used for further analysis.

### 3.4. Assessment of Plant Infestation by Pathogens

Plant infestation by pathogens was analyzed in 2017–2018 at the milk-dough stage (BBCH 77–83). Three uppermost leaves from 10 plants were taken for phytopathological analysis in each of the 4 repetitions. The aim of this analysis was to determine the percentage of leaf-blade surface damaged by specific pathogens. This method of assessment on leaf diseases was in line with the recommendations of the European and Mediterranean Plant Protection Organization (EPPO) [40].

### 3.5. Assessment of Fusarium spp. Occurrence

An assessment of the occurrence of *Fusarium* head blight was carried out at the milk-dough stage of wheat (BBCH 77-83). The set of 4 × 50 randomly selected ears from each experimental combination was analyzed to determine the percentage of their infestation. After the harvest, the grains were subjected to mycological analysis. From each combination, 4 × 100 grains were randomly selected. After rinsing with running water and decontamination for 2.5 min in 1% NaOCl, followed by rinsing three times with sterile water, the grains were placed into BD DIFCO™ PDA (Potato Dextrose Agar; Becton, Dickinson and Company, Franklin Lakes, NJ, USA) (pH = 5.5), where they incubated for 6 days in the temperature of 20 °C. The fungus growing colonies were subsequently grafted onto PDA slants, where they were identified using mycological keys [41].

### 3.6. Chemicals

Acetonitrile and methanol hypergrade (>98%) were analytically graded and bought from J.T. Baker (Deventer, The Netherlands). 2,2-diphenyl-1-picrylhydrazyl radical (DPPH^•^), 4-dodecylresorcinol, formic acid (LC-MS grade) and 5-*n*-pentadecylresorcinol were delivered by Sigma-Aldrich, (St. Louis, MO, USA). Ethyl acetate, *n*-hexane and acetone were purchased from Acros Organics BVBA (Geel, Belgium). Other reagents (*m*-hydroxybenzoic acid, sodium hydroxide, hydrochloric acid, ascorbic acid) were delivered by commercial suppliers, including Chempur (Piekary Śląskie) and Poland POCH S.A. (Gliwice, Poland). Using a purification system, Milli-Q water was obtained (Millipore Corp., Molsheim, France).

### 3.7. Chemical Analyses

#### 3.7.1. Phenolic Acids (PAs)

##### Extraction of PAs from Spring Wheat Samples

The grain was ground and defatted using the Soxhlet apparatus with hexane. In a falcon tube, 200 mg of grain was weighed and suspended in a 2% aqueous solution of ascorbic acid containing 20 µL of the internal standard 3-hydroxybenzoic acid. The hydrolysis with 4 M NaOH was performed in the dark for a period of 4 h. The resulting hydrolysate was acidified with 6 M HCl until the pH reached 2 and was then centrifuged for five minutes at 7000× *g* and 10 °C (Sigma, Osterode am Harz, Germany). The supernatant was extracted thrice via the liquid–liquid method with hydrated ethyl acetate. The extract was then evaporated to dryness on a vacuum evaporator and subsequently dissolved in 4 mL of 25% methanol. Samples with a concentration of 50 mg/mL were prepared for chromatographic analyses. For each cultivar, three separate samples were prepared.

##### Determination of PAs Using UPLC-DAD-MS

The content of PAs in the wheat extracts was determined by reversed-phase ultra-high pressure liquid chromatography (UHPLC), conducted on a Waters ACQUITY UPLC^®^ Systems chromatograph (Waters Corporation, Milford, MA, USA) equipped with a photodiode array detector and coupled to a triple-quadrupole mass spectrometer (Waters ACQUITY^®^ TQD, Micromass, Manchester, UK). The samples were separated on a Waters ACQUITY UPLC^®^ HSS C18 column (Milford, CT, USA, 1.0 × 100 mm; 1.8 μm) at a temperature of 30 °C. The mobile phase comprised two solvents: solvent A (0.1% formic acid in MilliQ water, by volume) and solvent B (0.1% formic acid in acetonitrile, by volume). The analytes were eluted using the following combination of isocratic and gradient steps: 0–0.07 min., isocratic 5% B; 0.07–8.33 min., 5–15% B; 8.33–8.67 min., 15–60% B; 8.67–9.33 min., isocratic 60% B; 9.33–9.40 min., 40–5% B; 9.40–12.00 min., isocratic 5% B. The sample injection volume was 1 μL. The detection of analytes was conducted in the negative ionization mode, utilizing a selected reaction monitoring method. The conditions of the MS analysis were previously published by Czaban et al. [42]. 

##### Antiradical Activity of PAs

The antioxidant activity of PAs in spring wheat cultivars was specified using a method described before by Kowalska et al. [29].

#### 3.7.2. Alkylresorcinols (ARs, Resorcinolic Lipids)

##### Extraction of ARs from Wheat Samples

The ARs (alkylresorcinols) fraction was extracted from non-defatted 1 g of each cultivar grain with 40 mL of acetone for 48 h at room temperature. Following centrifugation at 7000× *g* for 5 min at 10 °C (Sigma, Osterode am Harz, Germany) and evaporation to dryness at 40 °C under vacuum, the extract was dissolved in 1 mL of acetonitrile. Three independent replicates of each sample at 500 mg/mL were used for chromatographic analysis.

##### UPLC-PDA-MS/MS Analysis of ARs

The quantitative content of alkylresorcinols in cereal grains was determined as described previously by Kowalska et al. [17], with minor modifications to the PDA operating range (190–450 nm) and the UV wavelength of peak quantification (275 nm). The analyses were conducted in positive ionization mode using ESI-MS/MS.

##### Free Radical Scavenging Activity of ARs

The antiradical activity of alkylresorcinols in spring wheat cultivars was specified using a method previously developed and published for the first time in 2022 by Kowalska et al. [29].

### 3.8. Statistical Analysis

The obtained results were subjected to statistical analysis using a Statistica version 10 and 12 (Stat. Soft. Inc., Tulsa, OK, USA) software on the strength of a multivariate analysis of variance and post hoc analysis. An analysis of variation (ANOVA) was performed, where the experimental factors were years and cultivars (years × cultivars). The significance of differences was verified using Tukey’s test at *p* ≤ 0.05. Statistical calculations for the comparison of the abundance of grains colonized by *Fusarium* spp. were made by means of a frequency analysis–chi-square (χ^2^) test of concordance. 

## 4. Conclusions

It was confirmed that hulled cultivars showed a significantly lower susceptibility to the *Fusarium* spp. pathogen in comparison to modern cultivars of wheat. Nine phenolic acids and their derivatives (*p*-coumaric acid predominantly in husks compared to grain with high ferulic acid levels) were identified and determined in the ancient wheat cultivars, as was their their antiradical activity. In contrast to this, the primary homologues of the alkylresorcinols C21:0 and C19:0 are concentrated especially in wheat grains, while C25:0 and C21:0 were characteristic homologues in husks. Despite lower yields, the ancient wheat cultivars proved to be a rich source of antioxidants with high antioxidant activity beneficial for human and animal health and alkylresorcinols significant for the resistance of wheat cultivars against *Fusarium* and other fungal pathogens. In addition, the effect of the production system and environmental conditions in the year have been assessed. The results of the study demonstrated that the organic production system exhibited the highest antioxidant activity, which correlated with the phenolic acids and alkylresorcinols present in the wheat. 

## Figures and Tables

**Table 1:** Influence of the years, cultivars and production systems on the phenolic acid concentration and antiradical activity. The mean phenolic acids (µg/g of the grain), total phenolic acids concentration (µg/g of the grain) and antiradical activity (in relation to caffeic acid’s activity = 1.00) of four *T. aestivum* L. subsp. *aestivum* cultivars cultivated in different production systems.

PHENOLIC ACID	Production System (A)	Year (B)		Cultivar (C)				LSD_50_		
		2017	2018	Harenda	Kandela	Mandaryna	Serenada	(A)	(B)	(C)
Protocatechuic acid	ORG	1.58 b	1.74 a	1.75 a	1.75 a	1.46 c–f	1.68 ab	0.2	0.2	0.2
	INT	1.22 c	1.75 a	1.63 a–c	1.60 a–d	1.27 f	1.45 c–f			
	CONV	1.27 c	1.63 ab	1.39 d–f	1.52 b–e	1.55 a–e	1.35 ef			
*p*-OH-Benzoic acid	ORG	4.42 b	7.21 a	6.14 b	4.48 d	5.53 bc	6.99 a	0.7	0.5	0.7
	INT	3.90 c	7.10 a	5.81 bc	4.52 d	4.75 d	6.94 a			
	CONV	3.93 c	6.90 a	5.16 cd	4.52 d	5.15 cd	6.83 a			
Vanillic acid	ORG	7.92 cd	9.48 b	8.22 b–d	9.10 bc	9.31 ab	8.18 b–d	1.3	1.1	1.5
	INT	7.20 d	9.15 b	7.92 cd	8.29 bcd	8.96 bc	7.53 d			
	CONV	8.08 c	10.77 a	8.77 b–d	9.45 ab	10.58 a	8.91 bc			
Caffeic acid	ORG	16.96 a	17.11 a	19.08 a	15.06 cd	18.15 ab	15.85 bc	2.3	1.9	2.5
	INT	12.14 c	12.17 c	13.00 d–f	10.21 g	12.00 fg	13.40 d–f			
	CONV	13.15 bc	14.16 b	14.44 c–e	13.30 d–f	12.58 ef	14.31 c–e			
Syringic acid	ORG	8.04 e	10.03 c	7.80 h	8.37 gh	9.97 cde	10.00 c–e	1.1	0.9	1.2
	INT	8.66 de	11.24 b	9.63 d–f	8.74 f–h	10.99 a–c	10.45 b–d			
	CONV	8.97 d	12.43 a	9.27 e–g	10.32 b–e	11.41 ab	11.80 a			
*p*-Coumaric acid	ORG	13.04 c	26.72 b	19.83 abc	19.44 abc	20.52 ab	19.73 a–c	6.3	5.2	6.9
	INT	10.07 c	31.00 a	25.20 a	17.74 bc	13.89 c	25.32 a			
	CONV	10.41 c	34.67 a	22.84 ab	22.15 ab	24.80 a	20.37 ab			
Ferulic acid	ORG	649.18 c	837.05 a	854.67 a	715.06 b–d	765.65 ab	637.07 c–e	117.6	97.7	129.6
	INT	525.43 d	700.24 bc	689.66 b–d	544.76 e	603.19 de	613.74 de			
	CONV	568.80 d	721.84 b	732.85 bc	636.00 c–e	553.09 e	659.33 b–e			
Sinapic acid	ORG	48.91 bc	58.98 a	67.06 a	40.28 ef	70.31 a	38.12 fg	8.4	7.0	9.3
	INT	46.63 c	46.8 c	54.46 b	31.57 g	56.88 bc	40.96 d–f			
	CONV	52.50 b	47.96 bc	65.28 ab	38.70 fg	48.03 de	48.92 cd			
Total	ORG	750.05 c	968.32 a	984.55 a	813.65 b–d	900.92 ab	737.62 c–f	130.1	108.1	143.4
	INT	615.25 d	819.46 bc	810.30 b–d	627.42 f	711.91 d–f	719.79 d–f			
	CONV	667.11 d	850.37 b	859.99 a–c	735.95 c–f	667.20 ef	771.82 b–e			
Antiradical activity	ORG	0.198 b	0.214 a	0.233 a	0.186 bc	0.221 ab	0.186 c	0.1	0.1	0.1
	INT	0.131 d	0.190 bc	0.179 c	0.146 e	0.159 d	0.158 d			
	CONV	0.141 d	0.195 b	0.198 b	0.154 d	0.157 d	0.165 cd			

Values belonging to the same traits marked by different letters in columns mean significant differences between production systems, and in rows for years or cultivars. ORG = organic, INT = integrated and CONV = conventional production system; significance at *p* ≤ 0.001.

**Table 2:** Influence of the years, cultivars and production systems on alkylresorcinol concentration and antiradical activity. Mean alkylresorcinols (µg/g of the grain), total alkylresoscinols concentration (µg/g of the grain) and antiradical activity (in relation to *α*-tocopherol’s activity = 1.00) of four *T. aestivum* L. subsp. *aestivum* cultivars cultivated in different production system.

Alkylresorcinol	Production System (A)	Year (B)		Cultivar (C)				LSD_50_		
		2017	2018	Harenda	Kandela	Mandaryna	Serenada	(A)	(B)	(C)
C17:0 + 19:1	ORG	66.66 c	80.13 a	84.01 a	70.46 c	72.97 c	66.12 d	1.4	1.0	1.8
	INT	65.87 c	74.63 b	73.31 c	71.16 c	77.69 b	58.86 e			
	CONV	64.73 c	80.17 a	80.67 ab	65.01 d	78.63 b	65.49 d			
C19:0	ORG	205.35 d	282.18 a	271.02 a	247.38 c	249.97 c	206.68 f	4.2	2.8	5.3
	INT	198.25 de	262.15 c	249.92 c	235.27 d	251.11 bc	184.49 g			
	CONV	194.20 e	272.64 b	263.38 a	219.69 e	262.39 ab	188.22 g			
C21:0	ORG	260.57 c	374.17 a	326.66 b	297.91 c	318.40 b	326.50 b	5.7	3.8	7.2
	INT	253.67 c	329.21 b	343.14 a	261.02 ef	284.75 cd	276.84 de			
	CONV	254.28 c	337.09 b	352.03 a	247.13 f	299.60 c	283.99 cd			
C23:0	ORG	37.67 d	71.68 a	59.91 a	45.35 c	55.52 a	57.93 a	2.7	1.8	3.4
	INT	36.92 d	60.25 c	61.39 a	45.26 c	42.72 c	44.98 c			
	CONV	37.81 d	65.26 b	60.96 a	43.00 c	47.61 bc	54.58 ab			
C25:0	ORG	11.42 c	25.29 a	20.80 b–d	21.77 a-c	18.06 cd	12.78 ef	1.7	1.1	2.1
	INT	12.97 c	19.54 b	21.36 a–d	16.57 de	16.82 de	10.28 f			
	CONV	12.81 c	26.04 a	17.00 c–e	23.24 ab	25.77 a	11.69 f			
Total	ORG	581.66 d	833.44 a	762.41 a	682.87 bc	714.92 b	670.01 c	11.5	7.7	14.6
	INT	567.69 d	745.78 c	749.13 a	629.27 d	673.10 c	575.45 e			
	CONV	563.84 d	781.20 b	774.03 a	598.07 de	713.99 b	603.98 de			
Antiradical activity	ORG	0.129 d	0.167 a	0.170 a	0.132 c	0.156 b	0.134 bc	0.1	0.2	0.1
	INT	0.127 d	0.146 c	0.159 ab	0.127 d	0.134 bc	0.126 d			
	CONV	0.129 d	0.154 b	0.152 b	0.125 d	0.157 b	0.133 c			

Values belonging to the same traits marked by different letters in columns mean significant differences between production systems, and in rows for years or cultivars. ORG = organic, INT = integrated and CONV = conventional production system; significance at *p* ≤ 0.001.

## Data Availability

Data are contained within the article and Supplementary Materials.

## Supplementary Materials

The following supporting information can be downloaded at https://www.mdpi.com/article/10.3390/molecules29174106/s1, Table S1: Yield (t/ha), ear planting (no/m^2^), 1000 kernel weight (g) of spring wheat cultivars cultivated in organic system. Table S2: Leaves infestation (% leaf area) of spring wheat cultivars by fungial pathogens in organic production system. Table S3: Mean phenolic acids (μg/g of the grain ± SD*), total phenolic acids concentration (μg/g of the grain ± SD*) and antiradical activity (in relation to caffeic acid’s activity = 1.00) of hull-less and hulled wheat cultivars cultivated in organic production system. Table S4: Total precipitation (mm) and average monthly temperatures (°C) in the field experiment. Table S5: Mean alkylresorcinols (μg/g of the grain ±SD*), total alkylresoscinols concentration (μg/g of the grain ± SD*) and antiradical activity (in relation to α-tocopherol’s activity = 1.00) of hull-less and hulled wheat cultivars cultivated in organic production system. Table S6: Selected elements of the agricultural practice of spring wheat in different crop production systems. Table S7: Plant protection products used in spring wheat cultivated in different production systems. Table S8: List of wheat cultivars tested.

## Abbreviations

Alkylresorcinols (ARs), 5-*n*-heptadecylresorcinol (C17:0), 5-*n*-nonadecylresorcinol (C19:0), 5-*n*-nonadecenylresorcinol (C19:1), 5-*n*-heneicosylresorcinol (C21:0), 5-*n*-tricosylresorcinol (C23:0), 5-*n*-pentacosylresorcinol (C25:0), sodium hydroxide (NaOH), sodium hypochlorite (NaOCl), hydrochloric acid (HCl), thin-layer chromatography (TLC), 2,2-diphenyl-1-picrylhydrazyl radical (DPPH^•^), ultra-performance liquid chromatography (UPLC), ultra-high pressure liquid chromatography (UHPLC), phenolic acids (PAs), photodiode array detector (PDA), mass spectrometry (MS), Biologische Bundesanstalt, Bundessortenamt und CHemische Industrie scale (BBCH scale), analysis of variance (ANOVA), honest significance difference (HSD), below the limit of quantification (LOQ), below the limit of detection (LOD), dry matter (DM), cultivar (cv.), standard deviation (SD), organic (ORG), conventional (CONV), integrated (INT), European and Mediterranean Plant Protection Organization (EPPO).
