# Supplementary material for: The Effects of a Cultivar and Production System on the Qualitative and Quantitative Composition of Bioactive Compounds in Spring Wheat (Triticum sp.)
Source: Molecules. 2024 Aug 29;29(17):4106. doi: 10.3390/molecules29174106 (PMC11397066; doi:10.3390/molecules29174106)
Supplement: Supplementary file 1 [file molecules-29-04106-s001.zip › molecules-3149698-supplementary.pdf]

SUPPLEMENTARY MATERIAL

The Effects of a Cultivar and Production System on the Qualitative and Quantitative Composition of Bioactive Compounds in Spring Wheat (*Triticum* sp.)

Iwona Kowalska [1,*], Sylwia Pawelec [1], Łukasz Pecio [1] and Beata Feledyn-Szewczyk [2]

[1] Department of Biochemistry and Crop Quality, Institute of Soil Science and Plant Cultivation-State Research Institute, Czartoryskich Str. 8, 24-100 Pulawy, Poland; spawelec@iung.pulawy.pl (S.P.); lpecio@iung.pulawy.pl (Ł.P.)
[2] Department of Systems and Economics of Crop Production, Institute of Soil Science and Plant Cultivation-State Research Institute, Czartoryskich Str. 8, 24-100 Pulawy, Poland; bszewczyk@iung.pulawy.pl
* Correspondence: ikowalska@iung.pulawy.pl; Tel.: +48-81-47-86-883

Table of Contents:

Table S1. Yield (t/ha), ear planting (no/m^2), 1000 kernel weight (g) of spring wheat cultivars cultivated in organic system.

Table S2. Leaves infestation (% leaf area) of spring wheat cultivars by fungial pathogens in organic production system.

Table S3. Mean phenolic acids (µg/g of the grain ± SD*), total phenolic acids concentration (µg/g of the grain ± SD*) and antiradical activity (in relation to caffeic acid's activity = 1.00) of hull-less and hulled wheat cultivars cultivated in organic production system.

Table S4. Total precipitation (mm) and average monthly temperatures (°C) in the field experiment.

Table S5. Mean alkylresorcinols (µg/g of the grain ± SD*), total alkylresoscinols concentration (µg/g of the grain ± SD*) and antiradical activity (in relation to *α*-tocopherol's activity = 1.00) of hull-less and hulled wheat cultivars cultivated in organic production system.

Table S6. Selected elements of the agricultural practice of spring wheat in different crop production systems.

Table S7. Plant protection products used in spring wheat cultivated in different production systems.

Table S8. List of wheat cultivars tested.

Table S1. Yield (t/ha), ear planting (no/m^2), 1000 kernel weight (g) of spring wheat cultivars cultivated in organic system.

	Species	Cultivar	Yield		Number of ears		1000 kernel weight		Yield	Number of ears	1000 kernel weight
			2017	2018	2017	2018	2017	2018	mean from 2017-2018		
Hull-less wheat species	*Triticum aestivum* L. subsp. *aestivum*	Goplana	4.31b **	4.43d	419bc	414bc	46.2bc	44.2c	4.37b	417bc	45.2d
		Harenda	3.88ab	3.92bc	327a	404bc	44.2b	39.5a	3.90ab	366ab	41.8b
		Kamelia	3.91ab	3.21a	370ab	296a	44.2b	45.0c	3.56a	333a	44.6cd
		Kandela	3.53ab	3.98bc	357ab	403bc	43.2b	38.4a	3.76ab	380ab	40.8a
		Mandaryna	3.30ab	3.98bc	344ab	420bc	39.5a	42.3b	3.64ab	382ab	40.9a
		Nimfa	4.48b	4.41d	402bc	442c	47.1bc	40.8b	4.45b	422bc	40.0a
		Rusałka	4.13ab	3.88b	426bc	453c	47.1bc	39.6a	4.01ab	440c	43.4c
		Serenada	3.95ab	3.95bc	444c	399b	49.4c	41.8b	3.95ab	422bc	45.6d
		Struna	3.74ab	4.07c	430bc	442c	45.2bc	43.5c	3.91ab	436c	44.4cd
		Zadra	3.20a	3.82b	387b	361b	44.0b	41.9bc	3.51a	374ab	43.0c
		Mean	3.82	3.97	391	403	45.0	41.7	3.90	397	43.4
Hulled wheat species *	*Triticum aestivum* L. ssp. *spelta*	Wirtas	1.97a	3.44b	330a	404bc	40.5a	41.9a	2.71a	367ab	41.2ab
	Triticum dicoccum	Płaskurka biała	2.65a	3.01ab	330a	388b	40.6a	44.1b	2.83a	359ab	42.4bc
		Płaskurka ciemna	2.50a	2.77a	358ab	438c	39.9a	42.3a	2.64a	398b	41.1ab
	Triticum monococcum	Samopsza	2.74a	3.06ab	470c	496d	39.5a	42.4a	2.90a	483d	41.0ab
		Mean	2.47	3.07	372	431	40.1	42.7	2.77	402	41.4

*Yield and weight of one thousand grains of hulled grain, approximately 50% is husk.

** Different letters after the mean values indicate significant differences ($p \leq 0.05$). For significant model effects a post hoc Tukey's honest significance difference (HSD) test was performed to compare mean values.

Table S2. Leaves infestation (% leaf area) of spring wheat cultivars by fungial pathogens in organic production system.

	Species	Cultivar	*Puccinia recondita*		*Septoria spp.*		*Drechslera tritici-repentis*		*Puccinia striiformis*	
			**2017**	**2018**	**2017**	**2018**	**2017**	**2018**	**2017**	**2018**
Hull-less wheat species	*Triticum aestivum* L. subsp. *aestivum*	Goplana	0.63a	4.50ab*	0.00a	0.00a	17.80cde	0.12a	1.03ab	0.00
		Harenda	0.77a	1.50ab	0.00a	1.25ab	25.90ef	0.12a	0.33ab	0.00
		Kamelia	0.33a	30.0cd	0.00a	2.62ab	19.77c-f	0.00a	0.87ab	0.00
		Kandela	0.23a	11.25bc	0.47a	3.87bc	0.47a	0.12a	0.8ab	0.00
		Mandaryna	0.93a	3.00ab	0.00a	3.12abc	27.80f	4.00a	0.33ab	0.00
		Nimfa	0.23a	5.75ab	0.00a	1.00ab	18.73c-f	0.12a	0.57ab	0.00
		Rusałka	0.00a	26.25cd	0.00a	1.62ab	11.77bc	0.00a	4.10cd	0.00
		Serenada	0.00a	4.75ab	0.00a	2.00ab	15.57cd	0.25a	0.40ab	0.00
		Struna	0.07a	10.25bc	0.00a	1.37ab	21.5def	0.12a	0.50ab	0.00
		Zadra	0.73a	37.50d	0.00a	11.25c	24.03def	0.00a	4.93d	0.00
		Mean	3.92	13.48	0.05	2.81	18.33	0.47	1.39	0.00
Hulled wheat species	*Triticum aestivum* L. subsp. *spelta*	Wirtas	2.33b	12.50bc	0.00a	1.87ab	17.23cde	0.75a	1.87ab	0.00
	Triticum dicoccum	Płaskurka biała	0.93a	0.00a	0.27a	0.00a	5.20ab	0.00a	1.00ab	0.00
		Płaskurka ciemna	0.57a	0.00a	0.00a	0.00a	3.83ab	0.00a	2.20bc	0.00
	Triticum monococcum	Samopsza	0.00a	0.00a	0.10a	0.00a	2.00a	0.12a	0.00a	0.00
		Mean	0.95	3.12	0.09	0.47	7.06	0.22	1.27	0.00

*Different letters after the mean values indicate significant differences ($p \leq 0.05$). For significant model effects a post hoc Tukey's honest significance difference (HSD) test was performed to compare mean values.

Table S3. Mean phenolic acids (µg/g of the grain ± SD*), total phenolic acids concentration (µg/g of the grain ± SD*) and antiradical activity (in relation to caffeic acid's activity = 1.00) of hull-less and hulled wheat cultivars cultivated in organic production system.

Species	Cultivar	Year	Plant mate r-ial	Protocate - chuic acid	*p*-OH-Benzoi c acid	Vanillic acid	Caffeic acid	Syringic acid	*p*-Coumaric acid	Ferulic acid	Sinapic acid	Salicylic acid	Total	Antiradic al activity
Hull-less wheat species *Triticum aestivum* L. subsp. *aestivum*	Goplana	2017	grain	1.65 ± 0.01c-f	5.02 ± 0.14f-h	7.94 ± 0.52g-m	15.30 ± 0.25f-h	7.83 ± 0.43f-h	16.22 ± 0.24j-l	648.43 ± 12.95f-h	44.42 ± 1.03j-l	LOD	746.83 ± 13.98f-j	0.167e-h
		2018	grain	1.73 ± 0.04b-f	8.25 ± 0.39bc	9.50 ± 0.65b-f	17.50 ± 0.36cd	8.48 ± 0.29e-g	31.25 ± 0.59d-f	806.60 ± 37.71b-d	52.37 ± 3.74h-j	LOD	935.66 ± 42.81b-d	0.204cd
	Harenda	2017	grain	1.51 ± 0.04f-j	4.17 ± 0.08ij	6.68 ± 0.43l-n	16.03 ± 0.49d-g	6.08 ± 0.21i-l	11.57 ± 0.42kl	646.37 ± 21.64f-h	53.41 ± 1.20g-i	LOD	745.81 ± 23.71f-j	0.171e-g
		2018	grain	2.00 ± 0.11a	8.11 ± 0.51c	9.75 ± 0.49b-e	22.14 ± 0.89a	9.52 ± 0.46b-e	28.08 ± 1.86e-i	1062.98 ± 72.05a	80.71 ± 2.66c	LOD	1223.29 ± 78.85a	0.296a
	Kamelia	2017	grain	1.52 ± 0.02f-i	5.31 ± 0.08e-g	8.00 ± 0.30f-m	12.59 ± 0.30j-l	6.75 ± 0.13h-j	17.10 ± 0.34h-l	640.52 ± 18.80f-h	44.24 ± 0.47j-l	LOD	736.02 ± 18.71g-j	0.163f-j
		2018	grain	1.56 ± 0.09e-i	9.81 ± 0.36a	9.08 ± 0.13d-i	16.05 ± 0.87d-g	9.07 ± 0.90c-f	38.60 ± 14.09c-e	853.44 ± 34.95bc	42.44 ± 0.98k-m	LOD	980.06 ± 46.97bc	0.198d
	Kandela	2017	grain	1.68 ± 0.01b-f	3.82 ± 0.07jk	8.80 ± 0.06e-j	16.92 ± 0.05d-f	7.95 ± 0.41f-h	13.66 ± 0.46kl	705.39 ± 15.27d-g	40.12 ± 0.22lm	LOD	798.34 ± 15.6e-i	0.167e-h
		2018	grain	1.81 ± 0.21a-d	5.35 ± 0.10ef	9.40 ± 0.72b-g	13.19 ± 1.17i-k	8.80 ± 0.29d-f	25.23 ± 2.24f-j	724.73 ± 42.15d-f	40.45 ± 2.62lm	LOD	828.96 ± 44.13d-h	0.172ef
	Mandaryna	2017	grain	1.35 ± 0.04h-l	3.96 ± 0.07j	8.28 ± 0.40e-k	15.99 ± 0.36d-g	8.23 ± 0.38e-g	12.86 ± 0.37kl	532.71 ± 4.37i-l	57.50 ± 0.83f-h	LOD	640.87 ± 4.77j-l	0.152jk
		2018	grain	1.57 ± 0.18e-h	7.11 ± 0.35d	10.33 ± 1.12b-d	20.32 ± 1.11ab	11.71 ± 0.51a	28.19 ± 2.90e-h	998.60 ± 79.92a	83.13 ± 5.14c	LOD	1160.96 ± 76.35a	0.262b
	Nimfa	2017	grain	1.56 ± 0.05e-i	4.66 ± 0.06g-i	7.30 ± 0.17j-m	15.41 ± 0.15f-h	7.31 ± 0.22g-i	16.06 ± 0.12j-l	629.80 ± 28.26f-i	44.44 ± 1.29j-l	LOD	726.54 ± 29.9h-k	0.167e-h
		2018	grain	1.90 ± 0.04ab	8.91 ± 0.26b	9.49 ± 0.67b-f	21.46 ± 0.67a	10.12 ± 0.20b-d	34.81 ± 7.65c-f	888.17 ± 44.13b	56.94 ± 0.67f-h	LOD	1031.79 ± 36.51b	0.213c
	Rusałka	2017	grain	1.34 ± 0.03i-l	4.32 ± 0.17ij	7.19 ± 0.60k-n	14.76 ± 0.55g-i	7.35 ± 0.20g-i	13.72 ± 0.49kl	611.04 ± 25.76g-j	46.21 ± 1.10i-l	LOD	705.92 ± 28.24i-l	0.159g-k
		2018	grain	1.67 ± 0.03b-f	7.70 ± 0.11cd	9.44 ± 0.27b-g	21.29 ± 0.35a	10.24 ± 0.64bc	29.34 ± 4.53e-g	867.99 ± 29.72bc	60.71 ± 3.45fg	LOD	1008.38 ± 27.85b	0.215c

		Serenada	2017	grain	1.77 ± 0.04a-e	5.73 ± 0.05e	7.94 ± 0.03g-m	18.91 ± 0.34bc	9.89 ± 0.85b-d	14.06 ± 0.09j-l	712.26 ± 20.33d-g	44.61 ± 0.48j-l	LOD	815.16 ± 21.62e-i	0.172ef
			2018	grain	1.59 ± 0.08d-g	8.26 ± 0.29bc	8.43 ± 0.32e-k	12.80 ± 0.56j-k	10.10 ± 0.51b-d	25.39 ± 5.41f-j	561.88 ± 36.64h-k	31.63 ± 1.61n	LOD	660.08 ± 44.39j-l	0.155h-k
		Struna	2017	grain	1.13 ± 0.03l	2.88 ± 0.07lm	7.85 ± 0.10h-m	9.11 ± 0.04mn	8.31 ± 0.25e-g	8.88 ± 0.20kl	524.93 ± 9.50j-l	35.42 ± 0.92mn	LOD	598.52 ± 9.63lm	0.149k
			2018	grain	1.63 ± 0.03c-f	4.32 ± 0.14ij	6.50 ± 0.22mn	16.00 ± 0.60d-g	5.32 ± 0.23k-m	16.21 ± 4.03j-l	707.83 ± 18.57d-g	62.96 ± 3.21fe	LOD	820.77 ± 20.62e-h	0.163f-j
		Zadra	2017	grain	1.36 ± 0.05g-k	2.81 ± 0.05lm	5.71 ± 0.52n	12.52 ± 0.17j-l	4.84 ± 0.60lm	7.22 ± 0.35l	535.00 ± 3.65i-l	49.41 ± 0.90h-k	LOD	618.88 ± 3.34k-m	0.153i-k
			2018	grain	1.29 ± 0.04j-l	4.41 ± 0.14hi	9.27 ± 0.20c-h	10.81 ± 0.54lm	10.65 ± 0.44ab	18.64 ± 0.61g-k	692.06 ± 23.97e-g	39.64 ± 1.57l-n	LOD	786.76 ± 24.78f-i	0.148k
Hulled wheat species	*Triticum aestivum* L. ssp. *spelta*	Wirtas	2017	grain	1.24 ± 0.02kl	4.20 ± 0.08ij	5.76 ± 0.52n	8.27 ± 0.20n	6.69 ± 0.14h-k	6.06 ± 0.12l	449.64 ± 10.55l	42.62 ± 0.75k-m	LOD	524.47 ± 11.14m	0.147k
				husk	4.85 ± 0.07cd	14.39 ± 0.29d	33.47 ± 1.37e	34.64 ± 0.27c	34.52 ± 0.41cd	1395.47 ± 32.14d	1536.22 ± 77.96c	6.25 ± 0.33f	2.58 ± 0.02a	3062.40 ± 98.21c	0.723d
			2018	grain	1.68 ± 0.06b-f	7.25 ± 0.42d	8.08 ± 0.56f-l	12.12 ± 0.70kl	10.54 ± 0.54ab	16.80 ± 0.51i-l	780.16 ± 27.12c-e	60.99 ± 2.06fg	LOD	897.63 ± 31.21c-e	0.156h-k
				husk	4.71 ± 0.22cd	15.66 ± 0.13d	30.86 ± 1.40e	36.07 ± 0.60c	28.84 ± 0.73e	1986.91 ± 32.17bc	2206.37 ± 60.26a	9.11 ± 0.26ef	LOQ	4318.53 ± 90.93a	0.892a
	Triticum dicoccum	Płaskurka biała	2017	grain	1.26 ± 0.01kl	2.81 ± 0.04lm	7.72 ± 0.53i-m	12.33 ± 0.19j-l	4.22 ± 0.37m	68.95 ± 0.66b	486.23 ± 2.83kl	55.00 ± 0.87f-h	LOD	638.51 ± 2.99j-l	0.159g-k
				husk	2.66 ± 0.02e	11.87 ± 0.15e	30.73 ± 1.13e	21.86 ± 0.47e	31.14 ± 0.52de	1212.13 ± 10.81e	965.27 ± 27.37e	24.97 ± 0.20c	LOQ	2300.63 ± 17.45e	0.753c
			2018	grain	1.86 ± 0.04a-c	4.16 ± 0.05ij	10.89 ± 0.27b	16.20 ± 0.33d-g	5.81 ± 0.31j-l	58.60 ± 1.91b	667.05 ± 14.04fg	83.61 ± 2.29c	LOD	848.19 ± 17.7d-f	0.178e
				husk	5.03 ± 0.25c	21.13 ± 0.34c	45.12 ± 3.06d	25.34 ± 0.78d	44.12 ± 2.65a	2038.43 ± 39.02b	1766.19 ± 26.53b	15.54 ± 3.22d	LOQ	3960.91 ± 19.50b	0.833b
		Płaskurka ciemna	2017	grain	1.27 ± 0.03kl	3.13 ± 0.02l	10.64 ± 0.47bc	14.03 ± 0.19h-j	5.24 ± 0.33lm	109.85 ± 0.90a	509.49 ± 8.21j-l	69.62 ± 1.69de	LOD	723.27 ± 9.03h-k	0.166f-i
				husk	4.44 ± 0.04d	25.96 ± 0.26b	73.91 ± 3.68a	23.87 ± 0.23d	39.80 ± 0.77b	1401.89 ± 45.02d	1186.43 ± 22.52d	13.08 ± 0.11de	LOQ	2769.39 ± 44.49d	0.726d
			2018	grain	1.84 ± 0.09a-c	3.93 ± 0.23j	14.19 ± 0.81a	17.38 ± 1.01c-e	5.96 ± 0.19i-l	43.75 ± 1.74c	643.14 ± 52.23f-h	113.27 ± 9.06a	LOD	843.46 ± 64.57d-g	0.167e-h

			husk	5.66 ± 0.33b	26.32 ± 0.92b	57.99 ± 2.61c	22.09 ± 0.49e	35.57 ± 0.61c	1909.00 ± 51.52c	1712.77 ± 28.71b	30.68 ± 3.04b	LOQ	3800.08 ± 85.52b	0.878a
Triticum monococcum	Samopsza	2017	grain	1.27 ± 0.05kl	2.23 ± 0.13m	6.60 ± 0.12l-n	15.61 ± 0.38e-h	4.40 ± 0.49m	41.84 ± 1.67cd	500.41 ± 2.53kl	76.41 ± 1.57cd	LOD	648.76 ± 5.13j-l	0.165f-i
			husk	5.06 ± 0.04c	30.89 ± 0.61a	66.75 ± 3.24b	57.65 ± 1.07a	46.33 ± 1.26a	2187.45 ± 13.84a	1408.11 ± 74.38c	31.58 ± 0.33b	2.38 ± 0.03b	3836.20 ± 68.55b	0.835b
		2018	grain	1.36 ± 0.08g-l	3.25 ± 0.07kl	7.82 ± 0.18h-m	17.03 ± 1.06c-f	5.61 ± 0.45j-m	33.28 ± 0.53c-f	672.05 ± 23.27fg	99.69 ± 0.91b	LOD	840.09 ± 25.45d-g	0.172ef
			husk	6.54 ± 0.11a	31.28 ± 0.42a	58.11 ± 1.30c	52.81 ± 0.51b	37.25 ± 1.24bc	2257.26 ± 17.34a	1736.86 ± 29.01b	40.53 ± 0.72a	LOQ	4220.64 ± 44.68a	0.885a

* - different letter within a column indicates significant differences ($p < 0.05$); statistical analysis was performed separately for grain and husk.
LOQ- below the limit of quantification. LOD - below the limit of detection.

Table S4. Total precipitation (mm) and average monthly temperatures (°C) in the field experiment.

Month	Temperature			Precipitation		
	**2017**	**2018**	**long-term mean**	**2017**	**2018**	**long-term mean**
III	6.0	0.4	1.9	32.0	31.0	28.1
IV	7.6	13.6	8.1	65.0	30.0	42.0
V	13.6	17.2	13.8	62.0	59.0	55.0
VI	18.1	18.8	17.1	31.0	38.0	71.0
VII	18.6	20.7	18.6	109.0	112.0	78.2
VIII	19.6	20.7	17.8	96.0	28.0	67.3

Table S5. Mean alkylresorcinols (µg/g of the grain ± SD*), total alkylresorcinols concentration (µg/g of the grain ± SD*) and antiradical activity (in relation to *α*-tocopherol's activity = 1.00) of hull-less and hulled wheat cultivars cultivated in organic production system.

Species		Cultivar	Year	Plant material	C17:0+C19:1	C19:0	C21:0	C23:0	C25:0	Total	Antiradical activity
Hull-less wheat species	*Triticum aestivum* L. subsp. *aestivum*	Goplana	2017	grain	72.46±2.05ef	246.20±1.74e-g	286.61±1.88g-k	35.44±0.11i-k	5.16±0.23h-j	645.86±4.69hi	0.235gh
			2018	grain	102.60±5.07a	341.37±24.58a	412.67±26.58a-c	86.47±6.47bc	24.83±1.27ab	967.93±63.46a	0.304a
		Harenda	2017	grain	74.25±4.01c-e	255.88±1.36ef	220.41±1.34n	30.86±1.00jk	9.54±4.12d-j	590.96±5.60j	0.179k
			2018	grain	88.93±3.62b	316.10±10.12ab	429.97±6.30ab	85.63±1.91bc	30.90±2.51a	951.53±22.35ab	0.294b
		Kamelia	2017	grain	65.02±1.17g-i	230.29±0.49f-i	287.04±0.89g-k	40.81±0.81i-k	7.76±1.93f-j	630.92±3.41ij	0.232h
			2018	grain	94.83±0.15b	317.77±3.69ab	437.73±9.56a	99.07±0.21a	28.03±3.97a	977.43±8.38a	0.313a
		Kandela	2017	grain	59.96±2.59ij	233.96±5.05f-h	264.11±1.80i-m	36.58±0.49i-k	7.42±1.55f-j	602.03±2.68j	0.205i
			2018	grain	79.63±2.31cd	286.40±12.22cd	331.97±13.01ef	54.43±3.75gh	29.97±2.55a	782.40±33.69d-f	0.260de
		Mandaryna	2017	grain	66.97±0.41f-h	239.33±2.39fg	252.76±4.66k-n	30.47±0.98k	5.11±0.87h-j	594.63±5.76j	0.192j
			2018	grain	79.77±0.32cd	288.23±18.27cd	383.17±22.93cd	78.93±4.90b-d	23.73±1.57a-c	853.83±38.95cd	0.266cd
		Nimfa	2017	grain	81.04±3.15c	256.27±2.25ef	298.01±3.19f-j	37.74±0.27i-k	8.63±1.14f-j	681.69±4.47g-i	0.244fg
			2018	grain	95.10±1.77b	301.80±4.55bc	392.13±8.61b-d	75.90±0.78c-e	23.93±5.02a-c	888.87±13.62bc	0.293b
		Rusałka	2017	grain	61.66±2.23h-j	225.29±1.31g-i	290.08±1.78g-k	39.50±1.64i-k	11.81±1.38d-h	628.35±1.56ij	0.253ef
			2018	grain	71.23±1.55e-g	267.90±11.47de	383.60±12.79cd	73.30±2.61de	27.17±3.17a	823.20±29.01c-e	0.275c
		Serenada	2017	grain	56.92±1.06jk	202.64±0.65ij	302.03±2.60f-i	43.86±0.58hi	9.20±1.60e-j	614.65±3.28ij	0.233h
			2018	grain	72.17±1.89ef	237.97±7.65fg	351.57±5.34de	67.73±4.68ef	16.57±3.77c-e	746.00±10.82e-g	0.268cd
		Struna	2017	grain	70.37±0.72e-g	253.37±1.62ef	308.26±0.35f-h	41.61±0.33ij	13.03±0.95d-g	686.64±2.24g-i	0.237gh

			2018	grain	63.37±2.80h-j	235.67±11.65f-h	327.17±17.89e-g	67.53±1.98ef	27.27±5.14a	721.00±34.13f-h	0.289b
		Zadra	2017	grain	60.83±0.95h-j	225.52±1.15g-i	270.21±0.88h-l	37.09±0.32i-k	6.05±1.16g-j	599.70±0.56j	0.186jk
			2018	grain	63.07±3.20h-j	219.67±10.81hi	292.97±12.10f-k	55.03±1.63g	11.33±1.05d-i	642.07±25.27ij	0.252ef
Hulled wheat species	*Triticum aestivum* L. ssp. *spelta*	Wirtas	2017	grain	51.25±0.73k	157.49±1.39j	217.96±1.76n	35.34±2.58i-k	7.77±1.32f-j	469.79±1.88kl	0.143l
				husk	LOQ	4.73±0.34d	92.04±1.25a	7.91±4.71de	162.21±5.78b	266.89±2.27b	0.088c
			2018	grain	73.30±1.25d-f	229.40±6.92f-i	363.90±8.91de	86.30±3.51bc	29.20±2.42a	782.10±18.14d-f	0.267cd
				husk	6.00±0.26a	26.97±0.86b	179.00±19.34b	14.23±0.50cd	289.10±30.11a	515.30±44.81a	0.134a
	Triticum dicoccum	Płaskurka biała	2017	grain	30.94±0.66m	161.74±2.91j	257.52±3.59j-n	32.93±1.25i-k	2.16±1.36j	485.29±4.19k	0.148l
				husk	LOQ	4.03±0.39d	42.48±1.92d	5.70±2.26e	21.14±0.52c-e	73.36±4.31d	0.068d
			2018	grain	44.33±0.45l	208.80±1.23hi	385.50±5.31cd	81.43±2.89b-d	17.20±0.44b-d	737.27±7.42fg	0.286b
				husk	1.30±0.35b	23.70±2.00a	174.50±7.22b	18.20±0.70c	2.17±1.51de	219.87±6.69c	0.096bc
		Płaskurka ciemna	2017	grain	25.63±1.05m	127.97±1.54k	223.84±0.61mn	40.26±1.51i-k	4.00±0.59ij	421.71±1.56kl	0.147l
				husk	LOQ	15.41±0.20c	53.82±1.90d	14.23±1.83cd	9.27±0.52de	92.73±1.50d	0.042e
			2018	grain	17.20±2.44n	117.37±14.34kl	231.43±33.29l-n	62.13±2.52fg	8.13±0.25f-j	436.27±49.71kl	0.130m
				husk	1.53±0.32b	28.20±1.73b	117.87±6.16c	29.77±1.40b	48.30±4.61c	225.67±3.32bc	0.104b
	Triticum monococcum	Samopsza	2017	grain	12.14±0.93n	79.51±0.61m	227.08±1.91mn	66.33±0.75ef	14.81±0.37d-f	399.87±2.87l	0.118n
				husk	LOQ	2.44±0.23d	36.94±2.58d	11.80±1.48c-e	LOQ	51.18±3.12d	0.040e
			2018	grain	10.97±1.00n	99.47±11.77lm	243.13±33.70l-n	87.90±13.14b	14.50±3.86d-f	455.97±57.98kl	0.154l
				husk	LOQ	17.03±1.29c	126.17±5.20c	41.33±2.45a	32.27±3.91cd	216.80±7.77c	0.094c

* - different letter within a column indicates significant differences ($p < 0.05$); statistical analysis was performed separately for grain and husk.
LOQ- below the limit of quantification.

Table S6. Selected elements of the agricultural practice of spring wheat in different crop production systems.

	Crop production system		
**Specification**	**organic (ORG)**	**integrated (INT)**	**conventional (CONV)**
Crop rotation	1. potato<br>2. spring wheat + undersown crop clovers and grasses<br>3. clovers and grasses<br>4. winter wheat + catch crop (mustard)<br>5. mixture oat + vetch	1. potato<br>2. spring wheat<br>3. faba bean<br>4. winter wheat + catch crop (mustard)	1. winter rape<br>2. winter wheat<br>3. spring wheat
Soil tillage	Mouldboard ploughing		
Organic fertilization	Compost (30 t/ha) under potato+catch crop	Compost (30 t/ha) under potato+catch crop	Rape straw, winter wheat straw
Mineral fertilization (kg/ha)	According to the crop requirements, natural P+K fertilizers (42+60)	N (85) + P (55) + K (75)	N (140) + P (60) + K (80)
Herbicides	0*	1	2
Fungicides	0	1	1-2
Insecticides	0	1	1-2
Harrowing	0	1	0

* frequency

Table S7. Plant protection products used in spring wheat cultivated in different production systems.

Production system	Plant protection products		
	**Herbicides**	**Fungicides**	**Insecticides**
	2017		
Organic	–	–	–
Integrated	Mustang Forte 195SE-0.8 L/ha	Input 460EC-1.0 L/ha	Decis Mega-0.125 L/ha
Conventional	Mustang Forte 195SE-0.8 L/ha	Input 460EC-1.0 L/ha	Fury 100 EW-0.1 L/ha
		Delaro 325EC-1.0 L/ha	Decis Mega-0.125 L/ha
	2018		
Organic	–	–	–
Integrated	–	Tilt Turbo 575 EC-1.0 L/ha	Fury 100 EW-1.0 L/ha
Conventional	Chwastox Extra 300SL-3.0 L/ha	Tilt Turbo 575 EC-1.0 L/ha	Fury 100 EW-1.0 L/ha
	Mustang Forte 195SE-0.8 L/ha		–
Active ingredients of plant protection products used:	Mustang Forte 195 SE: aminopyralid-10 g/L, florasulam-5 g/L, 2.4D-180 g/L; Chwastox Extra 300SL: MCPA 300 g/L (26.5%)	Input 460EC: prothioconazole 175 g/L (15.68%), spiroxamine 150 g/L (13.44%); Delaro 325EC: prothioconazole 160 g/L (16.3%), trifloxystrobin 300 g/L (30.5%); Tilt Turbo 575 EC: propiconazole-125 g/L, fenpropidine-450 g/L	Decis Mega: deltamethrin-50 g/L Fury 100 EW: zeta-cypermethrin-100 g/L

Table S8. List of wheat cultivars tested.

Type of wheat	Wheat species	Wheat cultivars	Country of origin	Breeding company and registration year	Crop production system	
					Organic	Conventional and Integrated
Hull-less wheat species	Common wheat (*Triticum aestivum* L. subsp. *aestivum*)	Goplana	Poland	DANKO Breeding Company, 2015	x	
		Harenda		MHR Małopolska Breeding Company, 2014	x	x
		Kamelia		Strzelce Breeding Company, IHAR Group, 2005. Deleted from the Cobor register in 2020	x	
		Kandela		DANKO Breeding Company, 2010	x	x
		Mandaryna		DANKO Breeding Company, 2014	x	x
		Nimfa		Strzelce Breeding Company, IHAR Group, 2016	x	
		Rusałka		Strzelce Breeding Company, IHAR Group, 2016	x	
		Serenada		Strzelce Breeding Company, IHAR Group, 2015	x	x
		Struna		DANKO Breeding Company, 2013	x	
		Zadra		Strzelce Breeding Company, IHAR Group, 2005	x	

Hulled wheat species	*Triticum aestivum* L. ssp. *spelta*	Wirtas	Poland	University of Warmia and Mazury in Olsztyn, 2015	x
	Triticum dicoccum	Płaskurka biała	Poland organic farm	Not know, old	x
		Płaskurka ciemna	Poland organic farm	Not know, old	x
	Triticum monococcum	Samopsza	Poland organic farm	Not know, old	x
